# Exploring the Use of Activity Trackers to Support Physical Activity and Reduce Sedentary Behavior in Adults Diagnosed With Type 2 Diabetes: Qualitative Interview Study Using the RE-AIM Framework

**DOI:** 10.2196/60066

**Published:** 2024-12-30

**Authors:** William Hodgson, Alison Kirk, Marilyn Lennon, Xanne Janssen

**Affiliations:** 1 Department of Physical Activity for Health, School of Psychological Sciences and Health, University of Strathclyde, 16 Richmond Street, Glasgow, G1 1XQ, United Kingdom, 44 07753324172; 2 Department of Computer and Information Sciences, University of Strathclyde, Glasgow, United Kingdom

**Keywords:** type 2 diabetes, physical activity, sedentary behavior, Fitbit, activity tracker, My Diabetes My Way, RE-AIM framework, diabetes care, clinical care, thematic analysis, health promotion

## Abstract

**Background:**

The prevalence of type 2 diabetes in adults worldwide is increasing. Low levels of physical activity and sedentary behavior are major risk factors for developing the disease. Physical activity interventions incorporating activity trackers can reduce blood glucose levels in adults diagnosed with type 2 diabetes. The My Diabetes My Way website is a support and educational platform for people diagnosed with diabetes and health care professionals. Users of the My Diabetes My Way website can upload their Fitbit (Google Inc) activity data into the system but this is not presently being analyzed and used routinely within clinical care. Developers of the My Diabetes My Way system are planning to allow different makes of activity trackers to be integrated with the platform.

**Objective:**

This qualitative study aimed to explore (through the RE-AIM [reach, effectiveness, adoption, implementation, and maintenance] framework) views from adults diagnosed with type 2 diabetes and health care professionals on the integration of activity trackers into type 2 diabetes care.

**Methods:**

Overall, 12 adults diagnosed with type 2 diabetes and 9 health care professionals (4 general practitioners, 1 consultant, 2 diabetes nurses, 1 practice nurse, and 1 physical activity advisor) were recruited through social media and professional contacts. Semistructured one-to-one interviews were conducted. Abductive thematic analysis was undertaken, and main themes and subthemes were identified. The RE-AIM framework was used to evaluate the themes with respect to the wider use of activity trackers and the My Diabetes My Way platform within type 2 diabetes clinical care.

**Results:**

Overall, 6 main themes (awareness, access, cost, promotion, support, and technology and data) and 20 subthemes were identified. Evaluation using the 5 RE-AIM dimensions found that reach could be improved by raising awareness of the My Diabetes My Way platform and the ability to upload activity tracker data into the system. Effectiveness could be improved by implementing appropriate personalized measures of health benefits and providing appropriate support for patients and health care staff. Adoption could be improved by better promotion of the intervention among stakeholders and the development of joint procedures. Implementation could be improved through the development of an agreed protocol, staff training, and introducing measurements of costs. Maintenance could be improved by supporting all patients for long-term engagement and measuring improvements to patients’ health.

**Conclusions:**

Through this study, we identified how the reach, effectiveness, adoption, implementation, and maintenance of integrating activity trackers into adult type 2 diabetes care could be improved.

## Introduction

Type 2 diabetes mellitus is a noncommunicable disease. Worldwide it is estimated that 483 million adults (20‐79 years of age) are living with type 2 diabetes [1]. By 2045, this number is estimated to rise to 705 million [1]. Annually 6 million adults die, prematurely due to type 2 diabetes [1]. In the United Kingdom, 5 million adults have been diagnosed with type 2 diabetes and the care costs for the National Health Service (NHS) are approximately £12 billion (US $15 billion) each year [2]. Major risk factors for developing type 2 diabetes include low levels of physical activity and sedentary behavior [2]. Adults diagnosed with type 2 diabetes have been found to be less physically active and spend more time engaged in sedentary behavior than those without the disease [3]. Physical activity even at low levels of intensity and reducing sedentary behavior can improve blood glucose levels in adults diagnosed with type 2 diabetes [4-7].

Physical activity interventions involving the use of activity trackers have been shown to increase physical activity and reduce sedentary behavior in adults diagnosed with type 2 diabetes [8]. Activity trackers are technological devices designed to measure the users’ steps, distance moved, physical activity intensity, and sedentary behavior [9]. Fitbit (Google Inc) consumer activity trackers are a valid and reliable method of measuring physical activity (steps, distance walked, energy expenditure, physical activity intensity, and sedentary behavior). When compared with laboratory-based tests of physical activity, Fitbit activity trackers have been shown to have large significant correlation coefficients of between 96.5 and 99.1 [10]. Recent discussions have suggested that national physical activity guidelines should be formulated around activity tracker measured physical activity rather than self-reported data, which tends to over or underestimate the users’ activities [11].

My Diabetes My Way is a web-based support and educational platform for diabetes patients and their health care professionals. The website allows users to access their patient records including prescribed medication and blood glucose measurements. The My Diabetes My Way website includes basic physical activity advice for patients though this element of the system is used less than other content [12]. Since 2019, patients have been able to upload their Fitbit activity data into the My Diabetes My Way platform. The developers of this platform state that there is an appetite for the linking of further makes of activity trackers, mobile apps, and web-based tools [13]. Users of My Diabetes My Way have shown a desire for uploading physical activity data into the system from alternative commercial activity trackers and mobile apps [13]. However, very little is known about if and how patients and health care professionals use the activity trackers in combination with web-based systems like the My Diabetes My Way platform to support the patients’ physical activity and reduce their sedentary behavior. Increasing our understanding of potential barriers and facilitators to the use of activity trackers and technology such as the My Diabetes My Way platform from both patients and health professionals will enable future improvement and development of digital health platforms and technologies to improve the clinical care of adults diagnosed with type 2 diabetes.

One way to evaluate the use of activity trackers is by using the RE-AIM (reach, effectiveness, adoption, implementation, and maintenance) framework. RE-AIM is a planning and evaluation framework used to improve the adoption and sustainable implementation of a wide range of evidence-based interventions including health-related interventions. The main RE-AIM dimensions are reach, effectiveness, adoption, implementation, and maintenance [14]. Reach is defined as the absolute number, proportion, and representativeness of individuals participating in a given initiative. Effectiveness is the impact of an intervention on outcomes, including potential negative effects, quality of life, and health components. Adoption is the proportion, representativeness, and absolute number of organizational agents involved in the intervention. Implementation is set at an organizational level and how the program was delivered by staff. Maintenance is defined as the extent to which a program or policy becomes embedded in routine practice. At an individual level, maintenance is a measure of the long-term impact of an intervention over 6-months [15].

This qualitative study aimed to explore (through the RE-AIM framework) views from adults diagnosed with type 2 diabetes and health care professionals on the integration of activity trackers into type 2 diabetes care. This study highlights how the integration of activity trackers into the My Diabetes My Way platform and general type 2 diabetes clinical care can be improved.

## Methods

### Ethical Considerations

Ethical approval for this qualitative interview study was obtained from the ethics committee of the University of Strathclyde (approval number 2021). For the study, both adults diagnosed with type 2 diabetes and health care professionals were provided with web-based participant information sheets. Informed consent was demonstrated by the digital signing of a consent form. Participants electronically selected individual items in the digital form, corresponding to the paper consent form, in order to confirm they had read and agreed with each item. Their electronic signature was achieved by entering their allocated 4-digit identification number. The research team undertook a number of steps to ensure the security of the information collected. To ensure anonymity each participant was allocated a unique 4-digit identification number and all data were stored under this number. Any information stored by the research team was stored within the university-approved, password-protected encrypted storage sites. Participants involved in this study received no form of compensation for taking part.

### Participants

#### Adults Diagnosed With Type 2 Diabetes

Participants (n=12) were recruited through social media posts (via Facebook and Twitter). Recruitment inclusion criteria included adults aged 18+ years, diagnosed with type 2 diabetes, residing in the United Kingdom, and able to read and write in English. Exclusion criteria included any other type of disease not listed in the inclusion criteria and any people aged out with the defined age bracket. The study participant information sheet and consent form were uploaded into the secure Qualtrics survey system. A link to this form was emailed to participants and their consent was recorded by indicating “yes” on the Qualtrics consent form. Once consent was received, participants were emailed a link to a baseline questionnaire on the Qualtrics system. This questionnaire gathered demographics, educational level, activity tracker use, and who if anyone provides physical activity advice within the person’s clinical care.

#### Health Care Professionals

Participants (n=9) were recruited through university contacts (via email) and social media posts (via Facebook and “X”). Recruitment criteria included adults aged 18+ years, residing in the United Kingdom, and experience of type 2 diabetes health care or physical activity for health. The study participant information sheet and consent form were uploaded into the secure Qualtrics survey system. A link to this form was emailed to participants and their consent was recorded. Once consent was received, a link to a questionnaire on the Qualtrics survey system to collect information on demographic and job role characteristics was emailed to participants.

### Procedure

**Figure 1.**
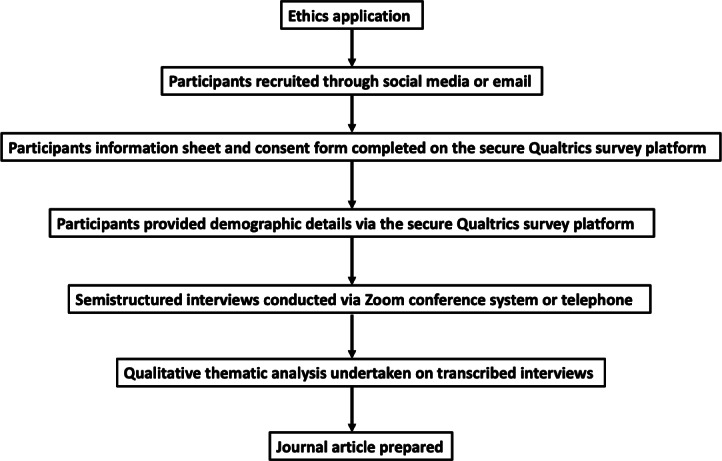
Flow diagram of study procedures.

A summary of the study procedures are provided in Figure 1.

#### Data Saturation

Initially, participant numbers were set at 15 for each group (type 2 diabetes participants and health care professionals). Data saturation was undertaken using the “stopping criterion” [15]. Participant interview saturation was reached when no new codes were identified [16]. All consented participants were retained with none dropping out.

#### Semistructured Interviews

One-to-one interviews were conducted with all participants. A semistructured interview schedule was prepared to focus on the 5 RE-AIM dimensions of reach, effectiveness, adoption, implementation, and maintenance. Interviews were conducted either over the telephone or via the secure Zoom conferencing platform. Each interview was recorded via a secure encrypted dictaphone. An abductive interview technique was used which started with the main RE-AIM dimension and worked down into the participants’ lived experiences in more detail [17]. Each interview was later transcribed verbatim from the dictaphone recording.

#### Analysis

NVivo 12 (Lumivero) software was used to manage the data and support the thematic analysis. All transcribed interviews were uploaded into this platform. A thematic analysis plan was developed which incorporated the 6-stage process recommended by Braun and Clarke [18]. The 6-stages are data familiarization (repeated reading of the transcripts), initial coding (identification of words or sentences connected to the analysis), initial development of themes (combining of codes into themes), review of identified themes, naming, and finalization of themes and preparation of the final journal article. The thematic analysis was conducted by the lead author (WH) and cross-checked by the 3 coauthors (AK, ML, and XJ). Differences in thematic and code names or meanings were resolved via discussion between these 3 researchers and the lead author.

## Results

### Participant Demographics

Participant demographics are displayed in Table 1.

**Table 1.** Participant demographics.

		Adults diagnosed with type 2 diabetes (n=12)	Health care professionals (n=9)
**Gender, n**			
	Female	8	5
	Male	4	4
Age (years), mean (SD)		53.17 (11.18)	52.57 (7.59)
Diagnosed with type 2 diabetes (years), mean (SD)		8.42 (6.76)	—^a^
**Education, n**			
	Degree	5	8
	Higher education	5	1
	School qualifications	2	0
**Ethnicity, n**			
	White	10	8
	Black	2	0
	Asian	0	1
**Country of residence, n**			
	Scotland	10	8
	England	2	1
**Home setting, n**			
	Rural	2	4
	Urban	10	5
**Used an activity tracker before the study, n**			
	Fitbit	3	—
	None	9	—
**Physical activity advice in clinical care, n**			
	General practitioner	2	—
	Diabetes nurse	5	—
	Practice nurse	1	—
	Diabetes consultant	1	—
	Cancer nurse	1	—
	None	2	—
**Role in clinical care, n**			
	General practitioner	—	4
	Consultant	—	1
	Diabetes nurse	—	2
	Practice nurse	—	1
	Physical activity advisor	—	1

a Not applicable.

### Findings

Abductive thematic analysis of interviews with adults with type 2 diabetes and health care professionals identified 6 main themes and 20 subthemes. These are displayed in Table 2 and discussed in more detail below.

**Table 2.** Thematic analysis of main themes and subthemes.

Main themes (n=6)	Subthemes (n=20)
Awareness	My Diabetes My Way Benefits of activity tracking Benefits to health
Access	Activity trackers Digital literacy barriers
Cost	Activity trackers Internet access Digital technology Health care staff
Promotion	Signposting Knowledge and credibility
Support	Advice Gym referrals Data interpretation Educational packages
Technology and data	Feedback Personalization Motivation Data management Fitbit functions

### Awareness

#### My Diabetes My Way

The majority of adults diagnosed with type 2 diabetes stated that they were not aware of the My Diabetes My Way platform and that it had not been discussed during consultations with a health care professional. For those registered on the platform, only one was aware of the ability to upload the Fitbit data into the system.

*I am not aware of this website…*

[Male, 61 years of age]

*I am registered but did not know you could upload your Fitbit activities.*

[Female, 44 years of age]

Health care professionals discussed awareness of the My Diabetes My Way platform and the ability to upload Fitbit data into the system. The majority of health care professionals were aware of the platform but none knew that users could upload the Fitbit data.

*I have limited knowledge of My Diabetes My Way and certainly did not know you could upload Fitbit data. This needs to be addressed by making staff aware.*

[Female, 57 years of age]

*I would say not aware. I have been using this system for a while and was not aware that Fitbit data could be uploaded.*

[Female, 61 years of age]

#### Benefits of Activity Tracking

Awareness of the benefits of tracking physical activity through activity trackers was discussed. Adults diagnosed with type 2 diabetes suggested that these devices would provide the user and health care professionals with an accurate indication of an individual’s physical activity.

*… monitoring a patients levels of activity. If these are low then they could be directed towards information on exercise or local classes.*

[Male, 37 years of age]

Health care professionals discussed the benefits of using activity trackers in clinical care. The ability to monitor a patient’s physical activity and provide appropriate advice and support was identified as the main benefit.

*The first would be to see how active a patient is. After that advice can be given on appropriate exercise activities which can then be further monitored.*

[Male, 52 years of age]

*The activity tracker could form part of a physical activity intervention which would include advice and attending exercise classes.*

[Male, 43 years of age]

#### Benefits to Health

Awareness of the benefits of using activity trackers to improve the health of users was explored. Adults diagnosed with type 2 diabetes stated that these devices could increase user’s physical activity and reduce sedentary behavior which would improve the health of the individual.

*It would give me a better understanding of how active I am and this may motivate me to do more physical activity and improve my overall health.*

[Female, 46 years of age]

Health care professionals discussed the benefits to a patient’s health if activity trackers were used in the clinical care of adults diagnosed with type 2 diabetes. Improved health was highlighted as the main benefit which would reduce the need for medical treatment.

*Long term this would benefit the patient’s health with less reliance on medication and treatment for related health conditions.*

[Male, 52 years of age]

### Access

#### Activity Trackers

Adults diagnosed with type 2 diabetes discussed how clinical care providers could gain access to activity trackers. It was suggested that these devices could be prescribed by the health care provider and used as part of a patient’s treatment plan.

*The tracker could be prescribed on the NHS especially for those on low incomes.*

[Female, 57 years of age]

Health care professionals discussed how patients could access an activity tracker as part of their clinical care. It was suggested that these devices could be prescribed by a health care professional.

*Prescribing an activity tracker could help motivate a patient to be more active.*

[Female, 61 years of age]

#### Digital Literacy Barriers

Access to the My Diabetes My Way platform was discussed during interviews with adults diagnosed with type 2 diabetes. It was suggested that accessibility could be improved by a health care professional providing clear guidance and instructions on how to register for this service, especially for people with limited information technology skills.

*I am not confident using computers and would need my GP or the practice nurse to provide me with support so I can register for this website.*

[Male, 61 years of age]

Health care professionals discussed the digital literacy barriers that may prevent a patient from accessing and using information technology as part of their clinical care. It was suggested that those with limited digital skills and confidence in using technology would need support and training.

*Some of my patients are uncomfortable using technology and would need support.*

[Male, 43 years of age]

### Cost

#### Activity Trackers

Adults diagnosed with type 2 diabetes discussed the costs with respect to purchasing an activity tracker. The majority of those interviewed said they would pay for a device if it was to form part of their type 2 diabetes care. If prescribed by a health care professional, the cost would be covered by the NHS though some suggested that with stretched budgets this may not be possible.

*I would be happy to pay for the activity tracker though not everyone can afford them.*

[Female, 72 years of age]

*Cuts in funding may prevent the NHS from giving these devices to patients.*

[Female, 46 years of age]

Health care professionals discussed the cost of purchasing an activity tracker. It was suggested that those who can afford the device should pay for it. If bought by the health care provider in bulk, the cost could be reduced for the patient. For those on a limited budget, the activity tracker could be prescribed.

*Our resources are stretched and those who can afford a Fitbit should pay for it. We could prescribe the Fitbit if the patient is on a low income.*

[Male, 43 years of age]

#### Internet Access

Adults diagnosed with type 2 diabetes discussed the costs associated with internet access. Those interviewed stated that they had to pay an internet provider for home access and that would be required to access their activity tracker data and the My Diabetes My Way platform. It was suggested that those on low incomes may need financial support to access the internet at home.

*Costs could also include accessing the internet.*

[Male, 61 years of age]

*Not everyone can afford internet access so they may need help to pay for this.*

[Female, 44 years of age]

Health care professionals discussed the cost implications of accessing the internet. It was suggested that those on limited incomes may need support with the costs. Poor internet coverage in rural areas could also increase access costs and make it difficult for people in these locations to use technology in clinical care.

*Not all of my patients can afford internet access.*

[Male, 43 years of age]

*Basic internet access is poor on the island. More efficient systems are expensive.*

[Female, 57 years of age]

#### Digital Technology

Adults diagnosed with type 2 diabetes discussed the costs associated with purchasing digital technology. They indicated that either a smartphone or computer would be needed to access activity tracker data and the My Diabetes My Way website. For those on low incomes, such devices could be prescribed by the NHS.

*Another cost would be buying a mobile phone or laptop. I already have these but some may struggle to afford them.*

[Female, 41 years of age]

Health care professionals discussed the costs associated with purchasing digital technology. It was highlighted that for patients to upload activity tracker data into the My Diabetes My Way platform they would need to have either a smartphone or computer. Patients on lower incomes may need support with the cost of these items.

*In addition to the activity tracker users would have to have a mobile phone or PC. Not all of my patients can afford these items.*

[Female, 61 years of age]

#### Health Care Staff

Adults diagnosed with type 2 diabetes discussed the cost implications with respect to health care staff if activity trackers are used as part of type 2 diabetes treatment. The main costs identified included staff training and staff time.

*This will be their time and training on how to use the activity tracker.*

[Female, 72 years of age]

Health care professionals discussed the costs associated with being health care professionals. The main costs identified were staff time and training.

*The main costs for our practice would be our time and any training to improve or knowledge and understanding.*

[Male, 53 years of age]

### Promotion

#### Signposting

Adults diagnosed with type 2 diabetes discussed the promotion of the My Diabetes My Way platform and how this could be signposted. Awareness of the system was low and it was indicated that better signposting by health care professionals was required. Suggested methods of communication were face-to-face consultations, posters in health service clinics, leaflets, SMS text messages, telephone calls, and emails.

*During a face-to-face consultation with a medical practitioner.*

[Female, 57 years of age]

*Other methods of promotion could be a telephone call from my diabetes nurse or an information leaflet when I attend the clinic.*

[Female, 41 years of age]

Health care professionals discussed the promotion of My Diabetes My Way and the ability to upload activity tracker data into the system. It was suggested that signposting should be the responsibility of the health care provider and make all staff aware. Promotion should be undertaken during face-to-face consultations between the patient and health care professional. Staff could be made aware during team meetings and work emails.

*I would prefer to promote these during my consultation with the patient.*

[Male, 42 years of age]

*I would make all our practice staff aware during team meetings or email.*

[Female, 57 years of age]

#### Knowledge and Credibility

Adults diagnosed with type 2 diabetes discussed the importance of the knowledge and credibility of health care staff when promoting the use of technology in clinical care. In recommending such technology, the health care staff should understand how it works, the benefits, and the ability to fully support the patient.

*The health care professional promoting should fully understand how the technology works.*

[Male, 37 years of age]

*The doctor or nurse should highlight the benefits of the technology during consultations with the patient.*

[Female, 47 years of age]

Health care professionals discussed the importance of health care professionals being credible and knowledgeable of any technology promoted. It was suggested that health care staff would need appropriate training to gain a full understanding before discussing and promoting information technology to a patient.

*I would need to ensure that we all had necessary training before discussing with a patient.*

[Male, 42 years of age]

### Support

#### Advice

Adults diagnosed with type 2 diabetes discussed the advice and support patients may require when using an activity tracker or the My Diabetes My Way platform. The main advice focused on individuals who possess low information technology skills and confidence in using such systems. Further advice in relation to exercise and physical activity was suggested.

*I am not confident using computers and would need plenty of advice on how to use them.*

[Female, 72 years of age]

*More advice on exercise and weight which I have not received during my treatment.*

[Female, 57 years of age]

Health care professionals discussed the advice patients may require to support them when using an activity tracker and the My Diabetes My Way platform as part of their type 2 diabetes treatment. The suggested advice included how best to access and use the technology.

*Probably like my gym work sitting down with the patient and talking them through the process, identifying their needs and encouraging them.*

[Male, 43 years of age]

*Many of my elderly patients would need advice to support them using these systems.*

[Female, 62 years of age]

#### Gym Referrals

Adults diagnosed with type 2 diabetes discussed the additional support they would need to compliment the use of activity trackers as part of their type 2 diabetes care. The majority stated that being prescribed a gym referral would motivate them to be more physically active.

*It would be nice to get directed to specific exercise classes for those diagnosed with type 2 diabetes.*

[Female, 46 years of age]

Health care professionals discussed the prescription of gym referrals to support the use of activity trackers in clinical care. The majority of health care professionals stated that prescribed gym referrals were already used to support adults diagnosed with type 2 diabetes. Some suggested that the gym referral through an exercise professional should incorporate an input about activity trackers.

*I already refer patients to the local gym. The fitness advisor would be the best person to show the patient how to use the activity tracker.*

[Female, 61 years of age]

#### Data Interpretation

Adults diagnosed with type 2 diabetes discussed the support patients may need to interpret the data collected on an activity tracker. It was suggested that health care professionals with knowledge of exercise and activity trackers should conduct the interpretation and communicate this to the patient.

*I would suggest a dedicated member of staff with knowledge of activity trackers and exercise. At the moment activity advice is limited and only occasionally discussed. The main focus is on medication and diet.*

[Female, 56 years of age]

Health care professionals discussed the support patients would need to interpret the data collected from the activity tracker. It was suggested that this support would be best delivered by a qualified health care professional with knowledge of physical activity and exercise.

*Ideally our health authority would employ fitness instructors.*

[Male, 53 years of age]

#### Educational Packages

Adults diagnosed with type 2 diabetes discussed the support in the form of educational packages that could be developed and deployed to assist patients to use activity trackers and the My Diabetes My Way platform effectively. It was suggested that the packages could be delivered online or booklet or face-to-face educational class.

*Some type of educational support package would assist patients to use technology in an effective manner.*

[Male, 37 years of age]

Health care professionals discussed patient support in the form of educational packages. It was suggested that these packages could be self-read or delivered in a classroom setting.

*Additional support packages could be produced or we could run special classes to support the patient.*

[Female, 61 years of age]

### Technology and Data

#### Feedback

Adults diagnosed with type 2 diabetes discussed how feedback from activity tracker data could be communicated and by who. The majority suggested the feedback should be delivered during a face-to-face consultation with a health care professional. Some proposed that when activity data was uploaded into the My Diabetes My Way platform the system interpreted the information and provided immediate feedback and advice.

*I would prefer my GP or the practice nurse to give me feedback from my activity data.*

[Female, 57 years of age]

*I have uploaded my Fitbit data onto MY Diabetes My Way. It would be great if the system would give me advice when I do this.*

[Female, 41 years of age]

Health care professionals discussed how best feedback from technology can be communicated to the patient. It was suggested that in the majority of cases this would be best served during face-to-face consultations. With respect to the My Diabetes My Way platform, participants proposed that the system analyzes the uploaded Fitbit data and provides feedback directly to the patient.

*Most patients would prefer feedback delivered by a health care professional.*

[Female, 62 years of age]

*Would it be possible for the website to feedback on the Fitbit information.*

[Male, 43 years of age]

#### Personalization

Adults diagnosed with type 2 diabetes discussed how data collected from an activity tracker and interpreted should be personalized for the user during the feedback process. This should take into account the patients’ medical history and understanding of physical activity.

*I would like any feedback to be personalised for my needs.*

[Female, 44 years of age]

Health care professionals discussed the personalization of data obtained through technology. It was suggested that as each patient has differing needs and goals the collected data should be personalized for the individual.

*After analysis I would personalise the feedback for the patient.*

[Female, 61 years of age]

#### Motivation

Adults diagnosed with type 2 diabetes discussed the motivational aspect of using data from technology in clinical care. Some suggested that activity trackers could motivate users to be more physically active. Before there, use participants said that users must be motivated to engage with the technology.

*My Fitbit has certainly motivated me to be more active.*

[Female, 41 years of age]

*Before using an activity the user must be motivated to engage with it.*

[Male, 61 years of age]

Health care professionals discussed how activity trackers could act as a motivational tool for patients. For this to be effective, it was highlighted that the patient would need to engage with the intervention for this to be successful.

*I can see these devices motivate some people to be more active. Saying that the patient always need to engage with any treatment plan.*

[Male, 52 years of age]

#### Data Management

Adults diagnosed with type 2 diabetes discussed who should manage the data obtained from activity trackers and stored on the My Diabetes My Way platform. All suggested that a health care professional such as the patient’s doctor, practice nurse, and diabetes nurse should have responsibility for managing the storage and use of the data.

*This would be my GP or the practice nurse.*

[Male, 37 years of age]

Health care professionals discussed the management of any data collected from patients. It was suggested that this must follow national guidelines and policies for health care organizations. Such data should be managed by the local health authority.

*Any data collected from patients must be stored and managed as per NHS policy.*

[Female, 61 years of age]

#### Fitbit Management

Adults diagnosed with type 2 diabetes discussed the available activity tracker functions. For those with knowledge of these devices, the preferred functions were daily steps, distance moved, challenges, and sleep.

*For me it is daily steps and distance travelled. Sleep is also interesting though I don’t bother too much about it unless I have a poor night’s sleep.*

[Female, 61 years of age]

*I enjoy the challenges as these motivate me to keep going. I can do these with friends and family.*

[Female, 57 years of age]

Health care professionals discussed the main activity tracker functions that could be used to support patients. The main functions identified were steps, physical activity intensity, distance walked, and stairs climbed.

*As a gym instructor I am aware of the useful functions. These would be steps taken, the level of physical activity, the distance moved and the height climbed.*

[Male, 42 years of age]

## Discussion

### Overview

This qualitative study aimed to explore (through the RE-AIM framework) views from adults diagnosed with type 2 diabetes and health care professionals on the integration of activity trackers into type 2 diabetes care. The study themes are discussed in alignment with the 5 main RE-AIM dimensions (reach, effectiveness, adoption, implementation, and maintenance) [14]. Some of the identified themes cross over more than one dimension. This evaluation seeks to identify how activity trackers can be implemented and effectively used by health care organizations to support the long-term maintenance of active lifestyles within type 2 diabetes care.

### Reach

In Scotland, 267,615 adults are diagnosed with type 2 diabetes, yet only 32,000 (12%) are presently active users of the My Diabetes My Way platform [19,20]. As reach is a measure of the proportion and representativeness of a health intervention the combined use of activity trackers together with the My Diabetes My Way platform should be made more visible and available to all adults diagnosed with type 2 diabetes [19]. This study has shown, for example, that awareness of the My Diabetes My Way platform was low among the adults diagnosed with type 2 diabetes though the majority of health care professionals did have knowledge of the system. We have also shown that there is low uptake and a lack of awareness of the ability to upload activity tracker data into the My Diabetes My Way platform. Reach could be significantly improved through better promotion of the platform and what it does and what the benefits are if people upload their tracking data. Previous research has shown that the implementation of web-based physical activity interventions for adults at risk of developing type 2 diabetes has only reached a small proportion of eligible patients and was not representative of the target population. Improved engagement strategies have been recommended by others to increase the level of awareness [21] and this study has shown this to be the case for both patients and professionals.

### Effectiveness

Effectiveness is a measure of the impact an intervention will have on important outcomes [14]. Previous research has shown that individuals who have uploaded their Fitbit data in the My Diabetes My Way platform have lower blood glucose readings, are less likely to develop diabetes foot problems, and are less likely to have experienced a myocardial infraction [21]. When the use of activity trackers is added as part of a type 2 diabetes physical activity intervention, HbA_1c_ levels have been shown to reduce, as have BMI and sedentary behavior [22]. This study has shown that people are interested in and motivated by the perceived and actual benefits of activity tracking. It would be extremely beneficial, therefore, to create better ways to link activity tracker data to recorded health outcomes and to physical activity guidelines on platforms such as My Diabetes My Way and make these features and their benefits much clearer to both patients and health care professionals [23].

Previous research has shown that personalized feedback via device-informed technology can increase levels of physical activity and reduce sedentary behavior in adults [24]. Our study also confirmed that physical activity feedback should be personalized for the individual patient. Our findings also indicate that to improve the effectiveness of these interventions further we would recommend that additional support should be made available to patients including advice, data interpretation, and educational packages. This level of personalization with supporting educational packages to the user will help overcome individual barriers such as digital literacy and also improve understanding of physical activity and sedentary behavior patterns to make interventions more inclusive and effective to a wider audience.

### Adoption

Adoption is the absolute number, proportion, and representativeness of settings and intervention agents (people who deliver the program) who are willing to initiate a program [14]. Our research identified that the integration of activity tracker data into the My Diabetes My Way platform and its use within type 2 diabetes clinical care was not being routinely adopted by health care providers. Research has shown that adoption can be enhanced through stakeholders working in close partnership [25]. We identified the main stakeholders as the My Diabetes My Way website developer, the NHS, regional health boards, local diabetes clinics, and local medical practices. In an effort to improve the adoption of activity trackers into type 2 diabetes clinical care, we recommend that stakeholders identify the added value of activity tracker use in terms of improved patient health and improve awareness for both patients and health care professionals. Furthermore, there is a need to develop and manage the organizational capacity by providing training to improve health care professional knowledge and understanding of implementing activity tracking into clinical care.

### Implementation

Implementation refers to the various stakeholders’ commitment to all aspects of an intervention’s protocol, including delivery consistency and the time and cost of the program [14]. At an individual level, implementation requires an understanding of how patients use the intervention [14]. Our research found that a protocol should be developed focusing on the implementation of activity tracker data into type 2 diabetes clinical care. We recommend that a protocol be produced which pays particular attention to organizational implementation and the development of health care staff promoting and delivering an activity tracker program. During the development of this protocol, intervention testing should be undertaken through the use of pilot studies [26].

Results in this study also show that health care providers are presently working with limited financial budgets. When implementing an activity tracker intervention health care organizations need to balance the costs against the health benefits. Our analysis identified costs such as the purchase of activity trackers, internet access, digital technology, and health care staff training. Research has shown that providing adult patients with a free-of-cost wearable activity tracker in combination with supporting technology can increase levels of physical activity and reduce sedentary behavior [27]. It would be useful to explore partnerships with commercial organizations and the opportunity to provide activity trackers at reduced or no cost. We also recommend further research and evaluation to understand how patients and health care professionals use activity trackers, the impact on health in the short and long terms, and further work to explore cost savings by comparing the intervention costs against any reduced health care costs.

### Maintenance

Maintenance is the extent to which a program or policy becomes routine practice within stakeholder organizations. At an individual level, the measure of maintenance is a patient’s engagement with the intervention for 6 or more months [14]. Research has shown that adults diagnosed with type 2 diabetes require long-term support and monitoring to maintain an active lifestyle after taking part in a physical activity intervention [28]. Personalized feedback and peer support have been shown to improve patient engagement, physical activity levels, and cardiorespiratory fitness of adults diagnosed with type 2 diabetes [29]. Many studies fail to address and assess the RE-AIM dimension of maintenance and as such few interventions last more than 6 months and fail to become routine clinical care [30]. Our study identified support factors to maintain patient engagement in an activity tracker intervention with the aim of becoming routine type 2 diabetes care. It is recommended that support factors such as prescribed gym referrals, patient assistance in interpreting activity tracker data, personalized data interpretation, and development of a personalized physical activity educational program be routinely incorporated into patient care.

Table 3 provides a summary of the main recommendations under each of the 5 main RE-AIM dimensions.

**Table 3.** Summary of main recommendations under the 5 main RE-AIM (reach, effectiveness, adoption, implementation, and maintenance) dimensions.

RE-AIM dimension	Main recommendations
Reach	Better promotion be undertaken through improved signposting Increase the knowledge and awareness of health care professionals
Effectiveness	Personalize physical activity feedback for the individual patient Additional support should be made available to patients including advice, data interpretation, and educational packages
Adoption	Stakeholders should identify the added value of activity tracker use (improved health), improving awareness (patients and health care professionals), and organizational capacity (health care professional knowledge and potential training) Development of joint procedures between stakeholders
Implementation	Development of an agreed protocol Development of a staff training program Introduce measurements of costs
Maintenance	Support all patients for long-term engagement Develop measures of improvements to patients’ health

### Strengths and Limitations

Through abductive thematic analysis, detailed main themes and subthemes were identified. Further evaluation of the results through the RE-AIM framework helped develop a better understanding of how the intervention could be improved and become routine practice within type 2 diabetes care. The sample size for this study was relatively small with 12 adults diagnosed with type 2 diabetes and 9 health care professionals though it was apparent that data saturation had been reached with similar responses suggested by participants within each group.

In conclusion, this study set out to explore through qualitative analysis the use of activity trackers to support physical activity and reduce sedentary behavior in adults with type 2 diabetes. Both adults with type 2 diabetes and health care professionals suggested that with amendments the use of activity tracker data could help support physical activity and reduce sedentary behavior in adults diagnosed with type 2 diabetes and this study has concluded with recommendations aligned to the RE-AIM framework on how to improve current implementation within both the My Diabetes My Way platform and general diabetes clinical care.

## Abbreviations

NHS: National Health Service
RE-AIM: reach, effectiveness, adoption, implementation, and maintenance
